# Bioengineering Materials in Dental Application

**DOI:** 10.1155/2017/2135036

**Published:** 2017-08-21

**Authors:** Wei-Jen Chang, Abe Shinichi, Shinji Kamakura, Ren-Yeong Huang, Jung-Wei Chen

**Affiliations:** ^1^School of Dentistry, College of Oral Medicine, Taipei Medical University, Taipei, Taiwan; ^2^Dental Department, Shuang-Ho Hospital, Taipei Medical University, Taipei, Taiwan; ^3^Department of Anatomy, Tokyo Dental College, Tokyo, Japan; ^4^Bone Regenerative Engineering Laboratory, Graduate School of Biomedical Engineering, Tohoku University, Sendai, Japan; ^5^National Defense Medical Center, Taipei, Taiwan; ^6^Department of Pediatric Dentistry, Loma Linda University School of Dentistry, Loma Linda, CA, USA

Bioengineering materials have been developed and applied in clinical therapy for rehabilitation of edentulous arches. The materials were also known with restoring the function and health in human beings. The concept for current bioengineering materials is based on a scaffold of tissue engineering triangle. Along with this evolution, bioengineering materials application is also accompanied with complications and failures.

This special issue aims to bring together state-of-the-art research contributions on the implants modification and applications to address the reduction of growing concerns after implant surgery. Potential topics of this special issue include UV photofunctionalization effect, mineral trioxide aggregate (MTA), antibody-mediated osseous regeneration (AMOR), computer-aided design (CAD) and 3D printing (3DP), bite force, inflammatory responses, bone healing, orthodontic power chains, and in vitro examination. After rigorous selection, 9 papers were elected.

In the paper “UV Photofunctionalization Effect on Bone Graft in Critical One-Wall Defect Around Implant: A Pilot Study in Beagle Dogs,” M.-Y. Kim et al. address UV photofunctionalization on the surface of implants that caused a large scale defects with bone graft but assists osseointegration and osteogenesis. UV-treated implant surface displayed enhancing osseointegration, such that UV treatment raised bone-to-implant contact being prone to new bone formation at the early stage, around 4 weeks.

In the paper “Cytotoxicity and Antimicrobial Effects of a New Fast-Set MTA,” M. Shin et al. propose the comparison of biocompatibility and antimicrobial effectiveness between the new Fast-Set MTA (FS-MTA) and ProRoot MTA (RS-MTA). These two endodontic procedure materials were tested to measure antimicrobial effect against bacteria. There is no cytotoxicity or bacterial inhibition observed by FS-MTA, comparing to RS-MTA.

In the paper “Collagen Sponge Functionalized with Chimeric Anti-BMP-2 Monoclonal Antibody Mediates Repair of Critical-Size Mandibular Continuity Defects in a Nonhuman Primate Model,” Y. Xie et al. report a chimeric anti-BMP-2 mAb mediated AMOR in clinically relevant mandibular continuity defect models. Anti-bone BMP-2 mAbs have been identified as an efficacious repair of bone defects. Cone beam computed tomography (CBCT) imaging and histologic detection confirmed de novo bone formation to suggest bone injury may be indispensable for AMOR. Thus, anti-BMP-2 mAb grabbed endogenous BMPs that triggered bone repair.

In the paper “Developing Customized Dental Miniscrew Surgical Template from Thermoplastic Polymer Material Using Image Superimposition, CAD System, and 3D Printing,” Y.-T. Wang et al. study the inflammation or other discomfort symptoms that occurred on placed customized miniscrews after surgery. To fabricate an accurate customized surgical template for dental orthodontic miniscrews, they used the techniques that integration of CBCT and laser scan image superposition, CAD and 3DP, were applied to. The customized miniscrew template showed well-fitting adaption and stable holding power.

In the paper “Influence of Deformation and Stress between Bone and Implant from Various Bite Forces by Numerical Simulation Analysis,” H.-C. Cheng et al. look at the influence of different bite forces that triggered deformation and stress on the bone and implant through 3D finite element (FE) methods. Near the implant neck in marginal bone is always accompanied with heavy stresses, revealing the physical force, including horizontal and vertical force, should be noted between implant and bone. Thus, to clinically maintain the structure and function of a bone and implant one should be aware of preserving.

In the paper “Fabrication of Novel Hydrogel with Berberine-Enriched Carboxymethylcellulose and Hyaluronic Acid as an Anti-Inflammatory Barrier Membrane,” Y.-C. Huang et al. point out a novel method established to fight against inflammatory responses. It has been well known that antiadhesion barrier membrane refers to a critical biomaterial for tissue protection from complications after surgery. A new hydrogel membrane composed of berberine-enriched CMC papered from bark of the* P. amurense* tree and HA (PE-CMC/HA) was fabricated in this study, named PE-CMC/HA, by mixing PE-CMC and HA as a base with the addition of polyvinyl alcohol to form a film. The result proved PE-CMC/HA membrane is a useful system for anti-inflammatory berberine release.

In the paper “Bone Healing Improvements Using Hyaluronic Acid and Hydroxyapatite/Beta-Tricalcium Phosphate in Combination: An Animal Study,” Y.-L. Chang et al. present the use of HLA as an aqueous binder of hydroxyapatite/beta-tricalcium phosphate (HA-*β*TCP) particles that decrease the amount of bone graft needed and elevate simplicity of operation in clinical situations. In rabbit, the calvarial bone defects were healed by HA-*β*TCP powder with HLA. Therefore, HLA addition promoted osteoconduction and also improved handling characteristics in clinical situations.

In the paper “Surface Treatment on Physical Properties and Biocompatibility of Orthodontic Power Chains,” H. C. Cheng et al. indicate the development of a surface treatment, nanoimprinting, for orthodontic power chains with alleviation of its shortcomings. After the surface treatment, the contact angle and the color adhesion of the orthodontic power chains became larger and less, respectively.

In the paper “Cytotoxicity of Titanate-Calcium Complexes to MC3T3 Osteoblast-Like Cells,” Y.-W. Chen et al. illustrate that monosodium titanates (MST) and MST-calcium (MST-Ca(II)) complexes had a cytotoxicity in MC3T3 osteoblast-like cells. Currently, it is found that MST suppressed MC3T3 cell metabolic activity in a dose-dependent manner; however, the mechanism of how MST and MST-Ca(II) played significantly cytotoxic role with regard to MC3T3 remains unknown. Thus, using the complexes remain with uncertain potential risks.

